# Safety and pharmacokinetics of ralinepag, a novel oral prostacyclin receptor agonist

**DOI:** 10.1016/j.jhlto.2025.100270

**Published:** 2025-04-23

**Authors:** Ali Ataya, James C. Coons, Natalie Patzlaff, Meredith Broderick, Scott Seaman, Namita Sood, M. Patricia George, Murali M. Chakinala

**Affiliations:** a Division of Pulmonary and Critical Care Medicine, University of Florida, 1549 Gale Lemerand Drive, Gainesville, FL 32610-3008; b University of Pittsburgh School of Pharmacy, 200 Lothrop Street, Pittsburgh, PA, 15213; c United Therapeutics Corporation, Research Triangle Park, 55 TW Alexader Dr. PO BOX 14186, Durham, NC, 27709; d University of California-Davis, 4150 V Street, PSSB Suite 3400, Sacramento, CA 95816; e National Jewish Hospital, 1400 Jackson Street, J231, Denver, CO 80206; f Division of Pulmonary and Critical Care Medicine, Washington University School of Medicine, 660 South Euclid Avenue, St. Louis, MO 63110

**Keywords:** Hypertension, Pulmonary/drug therapy, Receptors, Prostaglandin/agonists, Pulmonaryarterial hypertension, Ralinepag

## Abstract

**Background:**

Ralinepag is an oral, potent, highly selective prostacyclin receptor agonist and is in development for pulmonary arterial hypertension. Ralinepag was formulated as an immediate-release (IR) capsule, later modified to an extended-release (XR) tablet to optimize administration for once-daily dosing.

**Methods:**

This phase I study evaluated the pharmacokinetic (PK) profile and relative bioavailability of ralinepag XR. There were two cohorts of healthy participants (*n* = 12 each). One received a single dose of ralinepag IR (30 µg) followed by single ascending doses of XR (60, 120, and 180 µg), and the other received single ascending doses of selexipag (200, 400, and 600 µg). Participants titrated to the highest tolerated dose. There was a 7-day washout period between each dose.

**Results:**

The ralinepag XR formulation showed a reduced maximum observed concentration (C_max_), delayed time of maximum observed concentration (T_max_), and similar area under the curve compared with the IR formulation. Ralinepag XR demonstrated a gradual increase in plasma concentration over 8 to 12 hours, followed by a slow decline, with a half-life of 19-23 hours. In contrast, the active metabolite of selexipag (MRE-269) exhibited a sharp peak with a half-life of 9-10 hours. Ralinepag XR was well-tolerated by healthy volunteers, with 9 of 12 participants reaching the highest dose. Adverse events were qualitatively similar to oral prostacyclin class therapies.

**Conclusion:**

Ralinepag XR has a half-life suitable for once-daily dosing. The reduced C_max_ and delayed T_max_ contributes to lower peak-to-trough fluctuations and may provide favorable effects for sustained efficacy and improved tolerability.

## Background

Pulmonary arterial hypertension (PAH) is a rare, progressive disease that can lead to right ventricular failure and with unacceptably high mortality rates.^1^, ^2^ On a molecular level, PAH results from an imbalance of vasoactive mediators, including prostacyclin deficiency, which is a powerful driver of PAH pathology.^3^ Prostacyclins engage prostacyclin receptors to increase cyclic AMP (cAMP) levels, facilitating vasodilation and antiproliferative effects in smooth muscle cells.^3^

Parenteral prostacyclins are a mainstay of treatment for PAH and improve hemodynamics, which subsequently leads to a reduction in right ventricular afterload and may contribute to reverse remodeling of the right heart.^4^, ^5^ Continuous activation of prostacyclin receptors may be responsible for the efficacy and tolerability benefits observed with parenteral prostacyclin therapy; however, it is important to recognize that some patients refuse or may be inappropriate candidates for parenteral therapies.^6^, ^7^, ^8^ Parenterally infused prostacyclins activate several receptors, including DP1, EP2, and prostacyclin receptor (IP).^9^ An oral prostacyclin therapy with a pharmacokinetic (PK) profile that mimics parenteral infusion could be a helpful advancement in PAH.

Ralinepag is an oral, potent, and highly selective IP receptor agonist in development for PAH. Ralinepag does not require metabolic activation and demonstrates 30- to 50-fold selective binding affinity for the IP receptor over other prostacyclin receptors.^10^ By binding the IP receptor, ralinepag inhibits cellular proliferation by increasing cAMP levels in pulmonary artery smooth muscle cells from patients with PAH.^11^, ^12^ Additionally, ralinepag has vasodilatory effects.

Selexipag is the only approved oral IP agonist in both the USA and the EU for the treatment of PAH.^10^, ^13^, ^14^ Ralinepag is 6- to 8-fold more potent at increasing in vitro cAMP levels compared with the active metabolite of selexipag.^10^, ^13^, ^14^ Selexipag is dosed twice daily (BID) and titrated to the highest tolerated dose up to the maximum dose of 1600 µg BID. Ralinepag is fully titratable with no maximum dose limit.^15^, ^16^ Ralinepag was originally developed as an immediate-release (IR) capsule dosed BID and was later modified to an extended-release (XR) tablet dosed once daily. The XR formulation intends to mimic the stable PK of parenteral prostacyclin therapy.

Preclinical models demonstrated high plasma protein binding of ralinepag (99%) and 98% oral bioavailability in non-human primates.^10^ Additionally, nonclinical testing demonstrated that ralinepag did not inhibit major human cytochrome P450 (CYP) enzymes (CYPs 1A2, 2D6, 3A4, 2C8, 2C9, and 2C19).^10^ In contrast, selexipag, a CYP2C8 substrate, requires dosage adjustments when co-administered with CYP2C8 inhibitors (e.g., clopidogrel, leflunomide, and deferasirox), impacting its usage in certain populations.^17^ In a mass balance study, the majority of ralinepag (60.6%) was excreted unmetabolized (Data on file). Phase I and phase II studies have indicated a promising safety and efficacy profile for ralinepag.^15^, ^16^, ^18^ ADVANCE OUTCOMES (NCT03626688), a global, phase III, randomized, placebo-controlled mortality and morbidity event-driven study, is currently underway to evaluate the efficacy and safety of the XR formulation in patients with PAH.

This report describes the outcomes of a phase I study comparing the bioavailability, safety, and tolerability of ralinepag XR with its previous formulation (IR) and selexipag IR to support its once-daily dosing regimen.

## Methods

### Study participants

This phase I study evaluated the PK profile and relative bioavailability of single ascending doses of ralinepag XR. This study was conducted in accordance with the Declaration of Helsinki and good clinical practice and all relevant laws and institutional guidelines. The study protocol was approved by the independent ethics committee at the study site on February 10, 2017. All participants provided written informed consent, and all privacy rights of human subjects were observed. The study was conducted by Quotient Clinical, Nottingham, UK, a contract research organization.

Eligible participants were healthy adults between the ages of 18 to 45 years with a body mass index between 18 and 35 kg/m^2^. Females were required to be non-pregnant and non-lactating and agreed to use an adequate method of contraception. Exclusion criteria included a history of drug or alcohol abuse in the past 2 years, current smoker, or clinically significant abnormal biochemistry, hematology, or urinalysis at screening as judged by the investigator.

### Study design

The study was a single-center, open-label, fixed sequence, non-randomized study in two cohorts of healthy, fasted participants. Participants attended the study clinic for a screening visit up to 28 days before dosing. For each treatment period, participants were admitted to the clinic on the evening prior to dosing (day-1). Participants arrived the day before their first (lowest) dose in each cohort, fasted overnight (8 hours), received a single dose, and PK samples were collected at pre-dose and at 0.5, 1, 1.5, 2, 3, 4, 5, 6, 8, 10, 12, 16, 20, 24, 36, 48, and 72 hours after dosing. After a 7-day washout period, the process was repeated with the next highest dose. Each participant in Cohort 1 received each dose in a sequential manner over four treatment periods, with one dose every eighth day. Each participant in Cohort 2 received each dose in a sequential manner over three treatment periods on day one of each, one dose every eighth day. For both cohorts, a follow-up phone call took place 5 to 7 days after the final dose.

Ralinepag doses were selected based on initial studies indicating tolerance, and the doses of selexipag were chosen based on the prescribing information starting dose. Participants in Cohort 1 (*n* = 12) received a single dose of ralinepag IR (30 µg), then increased single doses of ralinepag XR (60, 120, and 180 µg) as tolerated. Participants in Cohort 2 (*n* = 12) received a single 200 µg dose of selexipag, then increased to a single dose of 400 µg and then a single dose of 600 µg as tolerated. The starting dose for selexipag was the standard clinical starting dose, and higher doses were given only if the lower dose was tolerated while staying below the maximum tolerated dose of selexipag.^14^ For each cohort, there was a minimum washout period of seven days between each dose. Participants were not allowed any medications for 14 days prior to the study, except for paracetamol, hormone replacement therapy, and hormonal contraception, and those deemed necessary by the investigator to treat adverse events (AEs).

### Safety assessments

The evaluation of safety parameters comprised analysis of AEs, laboratory variables (virology and follicle-stimulating hormone (post-menopausal female participants) at screening only; hematology, clinical chemistry, and urinalysis at screening and 24 hours post-dose), vital signs (blood pressure and heart rate at screening, pre-dose and 1.5, 4, 24, and 48 hours post-dose; oral temperature at pre-screening only), electrocardiogram (ECG; at screening, pre-dose and 1.5, 4, 24, and 48 hours post-dose), and physical examination (screening, pre-dose, and 48 hours post-dose). AEs and medications were coded using the Medical Dictionary for Regulatory Activities (MedDRA; v19.1) and the World Health Organization Drug Dictionary Enhanced (2016 version or more recent version), respectively. Subjects were withdrawn from the study for the following reasons: serious or severe AE (e.g., QTcF > 500 ms, abnormally high alanine aminotransferase concentration, etc.), non-tolerable AE (e.g., nausea or vomiting of moderate severity not responding to treatment), pregnancy, concurrent illness, at the discretion of the investigator, or at the subject’s request.

### Pharmacokinetics

The estimation of PK parameters by non-compartmental analysis methods was performed using Phoenix WinNonlin software (v6.3, Certara USA, Inc., USA). The following parameters were estimated for ralinepag and selexipag, and selexipag active metabolite (MRE-269): maximum observed concentration (C_max_), time of maximum observed concentration (T_max_), concentrations at 12 (C_12_) and 24 hours post-dose (C_24_), area under the curve from time zero extrapolated to infinity (AUC_0-inf_), area under the curve from time zero to last measurable concentration (AUC_0-last_), and apparent elimination half-life (T_1/2el_). The PK parameters underwent a natural logarithmic transformation and were analyzed using mixed- effect modeling techniques.

### Statistical analysis

Based on experience from previous similar studies, a total of 12 participants were enrolled in each cohort with the aim of 10 evaluable participants per cohort, although no formal statistical power calculation was done. Separate safety populations for Cohort 1 and Cohort 2 were determined and included all participants receiving at least one dose of study drug. The safety population was used for the analysis of demographic and baseline characteristics and all safety data. Summary statistics (i.e., mean, median, SD, CV%, minimum, maximum, and n) were calculated for plasma concentrations of ralinepag, selexipag, and selexipag metabolite for each time point and treatment using the PK population. Any participant that received at least one dose of an investigational agent was included in both the safety and PK populations.

Relative bioavailability (Frel) of ralinepag XR compared with IR was calculated using dose-adjusted parameters for AUC_(0-inf)_ and C_max_ using the following calculation: (Parameter[XR]/Parameter[IR]) x (Dose[IR]/Dose[XR]) x 100. Frel summary statistics were based on estimates calculated for individual subjects. Additionally, statistical analyses were performed on the PK parameters AUC_(0-last)_ and C_max_ to assess dose proportionality for Cohort 1 only. The PK parameters underwent a natural logarithmic transformation. The model included a term for dose fitted as a covariate. In addition, the power model was used to estimate the increase in the PK parameter resulting from a doubling in the dose.

## Results

### Demographics

There were 12 participants between 21 and 44 years of age enrolled in each cohort for a total of 24 participants (12 males and 12 females). One participant discontinued from selexipag (Cohort 2) at the investigator’s discretion because they were unable to be cannulated. Most participants were White 18 (75%), and 3 (13%), 2 (8%) and 1 (4%) were Black, Other, or Asian, respectively. There were no notable differences between cohorts in terms of demographic characteristics or lifestyle details.

### Pharmacokinetics

Following single-dose administrations, the ralinepag XR formulation has a reduced C_max_, an extended T_max_, and a similar AUC and half-life when compared with the IR formulation (Table 1), which may lead to less peak-to-trough fluctuation with repeat dosing. T_max_ values of ralinepag XR were delayed relative to ralinepag IR, indicating a slower absorption rate due to the slower tablet dissolution of the XR formulation. Maximum plasma concentrations (C_max_) for ralinepag IR occurred within 2 hours of dosing (T_max_ range 0.5-2 hours) and later for ralinepag XR (T_max_ range 2-10 hours and 2-16 hours) for the 60 and 120 µg dose, respectively, and at approximately 10 hours post-dose for the 180 µg dose (T_max_ range 4-24 hours). The extended T_max_ range at the highest dose is expected given the limited sample size and variability between individual participants. For dose-adjusted C_max_, ralinepag XR had lower peak exposure levels when compared with ralinepag IR. Dose-adjusted AUC_0-inf_ following 60 and 120 µg ralinepag XR was lower than ralinepag IR 30 µg, while 180 µg ralinepag XR was similar to ralinepag IR. Exposure to ralinepag XR increased with an increase in dose; formal statistical analysis indicated that the increase in C_max_, AUC_0-last,_ and AUC_0-inf_ was slightly higher than dose proportional over the 60 µg to 180 µg dose range.

**Table 1:** PK Parameters of Single Ascending Doses of Ralinepag IR, Ralinepag XR, and Selexipag Metabolite (MRE-269)

	Ralinepag IR	Ralinepag XR			Selexipag Metabolite (MRE-269)		
Parameter Units	retain-->30 µg (*N* = 12)	retain-->60 µg (*N* = 12)	120 µg (*N* = 12)	180 µg (*N* = 9)	200 µg (*N* = 12)	400 µg (*N* = 12)	600 µg (*N* = 5)
AUC_0-inf_ h*ng/ml	13.5 (42.6)^a^	23.2 (27.3)^b^	38.2 (35.8)^c^	83.1 (67.4)	24.7 (33.8)	53.8 (38.8)	67.5 (41.3)
AUC_0-last_ h*ng/ml	12.1 (45.1)	18.1 (40.9)	32.4 (36.6)	76.4 (63.8)	24.5 (32.4)	58.2 (41.2)	67.2 (41.6)
C_12,_ ng/ml	0.279 (39.9)	0.498 (35.5)	0.871 (36.8)	2.23 (70.1)	0.368 (49.7)	1.080 (59.9)	1.170 (73.1)
C_24,_ ng/ml	0.175 (62.2)	0.334 (51.6)	0.651 (44.0)	1.45 (73.3)	0.082 (77.7)	0.275 (78.0)	0.329 (137.5)
C_max_ ng/ml	1.16 (25.8)	0.727 (38.4)	1.33 (42.1)	2.83 (59.9)	4.33 (36.2)	8.83 (34.1)	10.5 (21.7)
C_max/_C_12_	4.16	1.46	1.53	1.27	11.8	8.18	8.97
C_max/_C_24_	6.63	2.18	2.04	1.95	52.8	32.1	31.9
T_max_ h	1 (0.5-2.0)	4.5 (2.0-10.0)	4.0 (2.0-16.0)	10.0 (4.0-24.0)	3.0 (1.5-4.0)	3.0 (2.0-4.0)	3.0 (2.0-3.0)
T_1/2 el_ h	20.95 (29.6)^a^	23.20 (34.8)^a^	19.22 (40.4)^a^	19.05 (24.3)	9.25 (87.6)	9.29 (54.4)	9.78 (26.7)
Frel AUC_0-inf_ %	NA	77.6 (24.8)^d^	62.7 (33.3)^c^	99.4 (54.9)	-	-	-
Frel C_max_ %	NA	31.3 (43.2)	28.7 (41.1)	42.0 (55.4)	-	-	-

Abbreviations: AUC_0-last_, area under the curve from time zero to the last non-zero concentration; AUC_0-inf_, area under the curve from time zero to infinity; C_max_, maximum observed concentration; C_24_, concentration at 24 hours; Frel, dose-adjusted relative bioavailability of ralinepag XR compared with the reference 30 µg ralinepag IR (Cohort 1 only); IR, immediate release; T_max_, time of maximum observed concentration; T_1/2__el_, apparent elimination half-life; XR, extended-release. All values are given as geometric mean and coefficient of variation (CV%), except Tmax given as median and range. Doses listed for selexipag metabolite represent the doses of the administered parent molecule. All participants were under fasted conditions.

a denotes *n* = 11,

b denotes *n* = 9,

c denotes *n* = 10,

d denotes *n* = 8.

**Figure 1:**
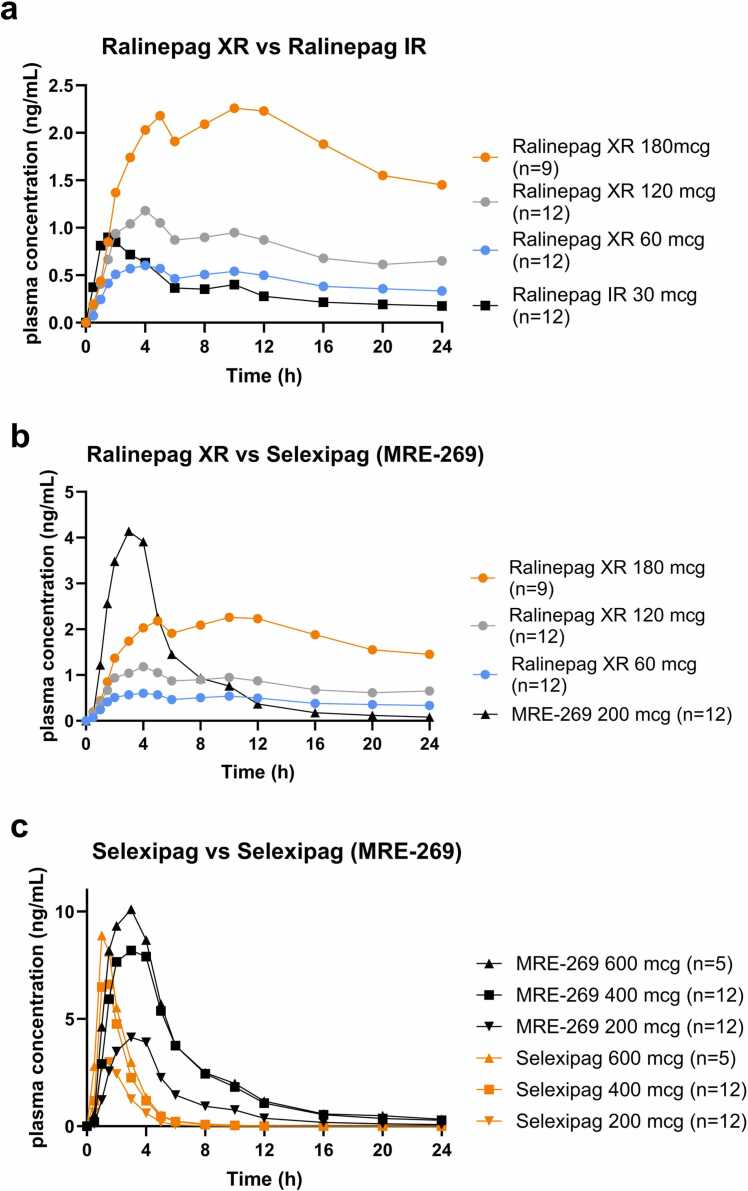
Ralinepag XR and selexipag pharmacokinetic profiles post-single dose. Slow release from the ralinepag extended-release (XR) formulation supports once-daily dosing for all concentrations, whereas, for selexipag, the plasma PK profile is consistent with a need for more frequent dosing. **(A)** Ralinepag XR versus ralinepag immediate release (IR), **(B)** Ralinepag XR versus selexipag metabolite (MRE-269), and **(C)** selexipag versus selexipag metabolite (MRE-269). The dosing for MRE-269 represents the selexipag parent drug and not the metabolite. All plasma concentrations are represented as geometric means.

Concentrations of ralinepag (IR and XR) and selexipag (selexipag and the active metabolite MRE-269) were detected in plasma by the first sampling time at 0.5 hour post-dose. Ralinepag XR shows a gradual increase in plasma concentration over the first 8 to 12 hours, followed by a slow decline. Higher doses resulted in higher peak concentrations (Figure 1a, b). In contrast, selexipag shows a rapid spike in concentration within the first 2 hours, followed by its active metabolite (MRE 269) at 4 hours with a decline of metabolite to near-zero levels by 20 hours (Figure 1b, c).

Following a single oral administration of 200, 400, and 600 µg selexipag, C_max_ occurred within 2 hours of dosing for all groups (T_max_ range 1-2 hours). The selexipag half-life was ∼1 to 1.5 hours. For MRE-269, C_max_ occurred by 3 hours for all groups (T_max_ range 1.5 to 4 hours) and the geometric mean half-life was approximately 9 to 10 hours post-dose. Geometric mean half-life for ralinepag XR was approximately 19 to 23 hours and appeared to be unaffected by dose. Slow sustained release of ralinepag XR supports once-daily dosing, whereas selexipag requires BID dosing (Figure 1).^13^, ^14^ For comparison, ralinepag XR, when examining the plasma concentration ratio between peak levels (C_max_) and “trough” levels at 24-hour (C_24_), we find the trough concentration is approximately half of the peak concentration across all doses. For selexipag, the active metabolite MRE-269 shows a peak-to-trough ratio (C_max_/C_12_) of about 8-12-fold difference after a 12-hour dosing interval.

### Safety

After single oral administration in Cohort 1, 9 of 12 participants reached the highest dose of ralinepag (180 µg XR), two did not progress to the optional final dose due to headache or nausea at the 120 µg XR ralinepag dose. All AEs were mild or moderate following ralinepag administration (Cohort 1). Overall, 11 out of 12 (92%) participants reported at least one grade 2 AE following dosing with single-dose ralinepag. There was a dose related trend for the number and severity of AEs with a lower prevalence reported following the 30 and 60 µg doses of ralinepag XR. The highest dose of ralinepag XR (180 µg), which was only offered to participants that tolerated the 120 µg dose, was not well tolerated because of gastrointestinal (GI) reactions and headache. The most commonly reported AEs overall were headache and dizziness, nausea, vomiting and diarrhea, and pain in extremity (Table 2). Of these, pain in the extremity only occurred following the highest dose of ralinepag XR.

**Table 2:** Summary of Treatment-Emergent Adverse Events for Single Ascending Doses of Ralinepag XR, Ralinepag IR, and Selexipag

	Cohort 1 Ralinepag, *n* (%)				Cohort 2 Selexipag, *n* (%)		
	IR 30 µg (*n* = 12)	XR 60 µg (*n* = 12)	XR 120 µg (*n* = 12)	XR 180 µg (*n* = 9)	200 µg (*n* = 12)	400 µg (*n* = 12)	600 µg (*n* = 5)
Patient reporting any SAE	0 (0)	0 (0)	0 (0)	0 (0)	0 (0)	0 (0)	0 (0)
Patient reporting any AE	3 (25.0)	5 (41.7)	10 (83.3)	9 (100.0)	6 (50.0)	9 (75.0)	4 (80.0)
Headache	2 (16.7)	2 (16.7)	10 (83.3)	9 (100.0)	4 (33.3)	7 (58.3)	3 (60.0)
Somnolence	0 (0)	0 (0)	0 (0)	0 (0)	0 (0)	1 (8.3)	1 (20.0)
Dizziness	0 (0)	0 (0)	1 (8.3)	2 (22.2)	0 (0)	0 (0)	0 (0)
Nausea	0 (0)	1 (8.3)	1 (8.3)	8 (88.9)	1 (8.3)	4 (33.3)	0 (0)
Vomiting	0 (0)	0 (0)	2 (16.7)	5 (55.6)	0 (0)	2 (16.7)	0 (0)
Diarrhea	0 (0)	1 (8.3)	0 (0)	3 (33.3)	0 (0)	0 (0)	0 (0)
Abdominal pain	0 (0)	0 (0)	0 (0)	1 (11.1)	0 (0)	1 (8.3)	0 (0)
Extremity pain	0 (0)	0 (0)	0 (0)	4 (44.4)	0 (0)	0 (0)	0 (0)
Jaw pain	0 (0)	1 (8.3)	0 (0)	0 (0)	1 (8.3)	2 (16.7)	2 (40.0)

Abbreviations: AE, adverse event; IR, immediate release; SAE, serious adverse event; XR, extended release. Participants in Cohort 1 received single ascending doses of ralinepag, starting with 30 µg of ralinepag IR, then progressing to ralinepag XR doses 60, 120, and 180 µg. Participants in Cohort 2 received single ascending doses of selexipag, starting with 200 µg, then progressing to 400 and 600 µg. For both cohorts, the highest dose in the sequence was optional, given the participant tolerated all previous doses. There was a minimum washout period of 7 days between each dose for both cohorts. The *n* refers to the number of participants experiencing the event.

Only 5 of 12 participants progressed to the highest dose level (600 µg) of selexipag, five did not progress due to intolerable AEs at the 400 µg selexipag dose or were withdrawn at the investigator discretion. One additional participant did not progress for logistical reasons, and another due to venous access problems. All AEs were mild or moderate following selexipag administration (Cohort 2). Overall, 11 of 12 (92%) participants reported at least one AE following dosing with selexipag. The most reported AEs were headache, nausea, vomiting, and pain in the jaw. There were no deaths, serious adverse events (SAEs), or other significant AEs reported during any part of this study for any drug.

## Discussion

These data are the first reports of PK for the ralinepag XR formulation. The ralinepag XR formulation had a delayed T_max_, and a lower C_max_ when compared with the ralinepag IR formulation. A reduced C_max_ may lead to better tolerability by reducing peak-to-trough fluctuations, which may mimic parenteral administration.^19^ When compared with the active metabolite of selexipag, ralinepag XR demonstrates a delayed T_max_, sustained concentrations over 24 hours, in addition to the known longer half-life of ∼19-23 hours making it more suitable for once-daily administration. Ralinepag XR was generally tolerated by healthy participants without any serious AEs.

Parenteral prostacyclins are considered the gold standard for the treatment of high-risk PAH.^20^ Meanwhile, currently available oral prostacyclin receptor agonists, while more convenient with less challenging administration than parenteral prostacyclins, have not demonstrated the same potency or efficacy.^20^, ^21^, ^22^ Ralinepag XR formulation may also be a future therapeutic option for patients transitioning from parenteral prostacyclins, although more research is needed. The mean half-life of ralinepag XR in this study was between 19 to 23 hours, consistent with prior studies.^15^ The only currently approved prostacyclin receptor agonist, selexipag, is dosed twice daily and capped at 1600 µg BID. Real-world evidence from the SPHERE registry of patients treated with selexipag shows that up to 32% of patients discontinued selexipag within the first 18 months of treatment because of an AE; and the overall duration of treatment for selexipag was 13.5 months (out of 18 months).^23^ Over 40% of SPHERE registry participants were in the high-dose strata (>1200 µg BID). Availability of a once-daily oral, potent, and continuously titratable, highly selective prostacyclin receptor agonist may lessen side effects while increasing efficacy and patient adherence.

The safety profile of ralinepag IR was supported with two phase I studies (single ascending dose and multiple ascending dose) that concluded no clinically significant safety issues.^15^ In this study, the AEs were dose-dependent, with more occurring at the higher doses of ralinepag XR (e.g., 180 µg). More participants tolerated ralinepag XR at a higher dose than selexipag, but the sample size was limited. Oral prostacyclins are typically up-titrated slowly, with tolerance increasing over time.^15^, ^18^ This was a single-dose escalation study that did not aim to evaluate dose titration to help overcome tolerability limitations, which is the standard treatment regimen for prostacyclins. The clinically efficacious dose and titration schedule for ralinepag has not been established, and ongoing clinical trials are being conducted to determine these. The ongoing phase III trials initiate ralinepag XR administration at 50 µg once daily and titrate weekly to the highest tolerated dose. Tablets strengths available for use in the phase III trials are 50, 250, and 400 µg, which differ from the doses studied in this phase I PK study. Preliminary research suggests that ralinepag does not interact with food.^24^

In a phase II study evaluating the efficacy and safety of ralinepag IR compared with placebo, the results showed the treatment group had a significant improvement in pulmonary vascular resistance and a numerical improvement in 6-minute walk distance (6MWD) in favor of the ralinepag group.^18^ The safety profile was further confirmed as tolerable and generally consistent with prostacyclin receptor agonists.^18^, ^20^ This study’s open-label extension demonstrated durable improvements in the 6-minute walk distance and a further reduction in pulmonary vascular resistance.^16^ Additionally, there was a notable decline in AEs after reaching and maintaining a stable dose.^18^ The ongoing phase III ADVANCE OUTCOMES (NCT03626688) and ADVANCE EXTENSION (NCT03683186) studies are designed to assess the efficacy and long-term safety of ralinepag XR when added to PAH standard of care or PAH-specific background therapy in patients with Group 1 PH. Future analyses of the PK population data from ADVANCE OUTCOMES will add to our understanding of repeated dosing for ralinepag XR. The study described here had some limitations. The study was conducted at a single center and involved a small number of healthy participants per cohort. Additionally, the total duration of treatment was constrained so that the safety of the healthy participants was not at risk. The dosing schedule used in this study of healthy volunteers does not simulate daily uninterrupted dosing as intended in patients with PAH. Ralinepag XR is currently undergoing further evaluation in the phase III studies, ADVANCE OUTCOMES and ADVANCE EXTENSION.

## Conclusions

The delayed T_max_ and sustained concentrations over 24 hours of ralinepag XR represents an optimized PK profile suitable for once-daily administration that should minimize differences between peak and trough levels. Ralinepag was generally well tolerated when administered under the conditions of the study, although there was individual variability of prostacyclin-related AEs when participants were escalated to higher doses. Availability of a once-daily oral, potent, and highly selective prostacyclin receptor agonist may lessen side effects, while increasing efficacy and patient adherence.

## Author Contributions

NP, SS, and MB created the initial draft with the support of a medical writer. Visualizations were created or revised by NP with input from all authors. All authors critically reviewed, edited, and approved the work for submission.

## Disclosure Statement

AA serves as clinical consultant for Savara. JC is a consultant to AstraZeneca, Bristol-Myers Squibb, and Janssen. NP, MB, and SS are current employees of United Therapeutics Corporation. PG is a site PI on the ADVANCE Outcomes study and is a speaker with United Therapeutic, she has also served on advisory boards and is on a steering committee with United Therapeutics, she is also the site PI for clinical trials sponsored by Janssen and Liquidia, she is a speaker with Janssen and Bayer, she has served on advisory boards for Merck, Gossamer, Janssen, and Liquidia, she serves as a volunteer on the Board of Directors for Team PHenomenal Hope, a nonprofit that is supported by United Therapeutics, Janssen, Gossamer, Merck, Bayer, and CVS Specialty Pharmacy. NS is a speaker with Boehringer Ingelheim and Bayer, serves on the consulting advisory boards for Merck and United Therapeutics Corporation, and receives funding from Bayer, Merck, United Therapeutics Corporation, Gossamer Bio, Respira Therapeutics, and Insmed. MC is a site PI for the ADVANCE Outcomes study and is a steering committee member for the United Therapeutic Corporation’s Jenesis Awards program; he also consults or sits on advisory boards for Bayer, Aerovate Therapeutics, Liquidia, Janssen, Merck, and Tectonic Therapeutic.

## Declaration of Competing Interest

The authors declare the following financial interests/personal relationships, which may be considered as potential competing interests: Ali Ataya reports financial support was provided by Savara Inc. James C. Coons reports financial support was provided by AstraZeneca PLC. James C. Coons reports financial support was provided by Bristol-Myers Squibb Company. James C. Coons reports financial support was provided by Janssen Pharmaceuticals Inc. Natalie Patzlaff reports financial support was provided by United Therapeutics Corporation. Scott Seaman reports financial support was provided by United Therapeutics Corporation. Meredith Broderick reports financial support was provided by United Therapeutics Corporation. Namita Sood reports financial support was provided by Boehringer Ingelheim Pharmaceuticals Inc. Namita Sood reports financial support was provided by Bayer Corporation. Namita Sood reports financial support was provided by Merck and Co Inc. Namita Sood reports financial support was provided by United Therapeutics Corporation. Namita Sood reports financial support was provided by Gossamer Bio Inc. Namita Sod reports financial support was provided by Respira Therapeutics Inc. Namita Sood reports financial support was provided by Insmed Inc. Patricia George reports financial support was provided by United Therapeutics Corporation. Patricia George reports financial support was provided by Janssen Pharmaceuticals Inc. Patricia George reports financial support was provided by Liquidia Corporation Inc. Patricia George reports financial support was provided by Bayer Corporation. Patricia George reports financial support was provided by Merck & Co Inc. Patricia George reports financial support was provided by Gossamer Bio Inc. Patricia George reports financial support was provided by Team Phenomenal Hope. Murali Chakinala reports financial support was provided by United Therapeutics Corporation. Murali Chakinala reports financial support was provided by Bayer Corporation. Murali Chakinala reports financial support was provided by Aerovate Therapeutics. Murali Chakinala reports financial support was provided by Liquidia Corporation Inc. Murali Chakinala reports financial support was provided by Janssen Pharmaceuticals Inc. Murali Chakinala reports financial support was provided by Merck & Co Inc. Murali Chakinala reports financial support was provided by Tectonic Therapeutic. If there are other authors, they declare that they have no known competing financial interests or personal relationships that could have appeared to influence the work reported in this paper.
